# Uncovering the immune microenvironment and molecular subtypes of hepatitis B-related liver cirrhosis and developing stable a diagnostic differential model by machine learning and artificial neural networks

**DOI:** 10.3389/fmolb.2023.1275897

**Published:** 2023-09-22

**Authors:** Shengke Zhang, Chenglu Jiang, Lai Jiang, Haiqing Chen, Jinbang Huang, Jieying Zhang, Rui Wang, Hao Chi, Guanhu Yang, Gang Tian

**Affiliations:** ^1^ Department of Clinical Medicine, School of Clinical Medicine, Affiliated Hospital of Southwest Medical University, Luzhou, China; ^2^ First Teaching Hospital of Tianjin University of Traditional Chinese Medicine, Tianjin, China; ^3^ National Clinical Research Center for Chinese Medicine Acupuncture and Moxibustion, Tianjin, China; ^4^ Department of General Surgery (Hepatobiliary Surgery), The Affiliated Hospital of Southwest Medical University, Luzhou, China; ^5^ Nuclear Medicine and Molecular Imaging Key Laboratory of Sichuan Province, Luzhou, China; ^6^ Academician (Expert) Workstation of Sichuan Province, Luzhou, China; ^7^ Department of Specialty Medicine, Ohio University, Athens, United States; ^8^ Department of Laboratory Medicine, The Affiliated Hospital of Southwest Medical University, Luzhou, China; ^9^ Molecular Diagnosis of Clinical Diseases Key Laboratory of Luzhou, Luzhou, China; ^10^ Sichuan Province Engineering Technology Research Center of Molecular Diagnosis of Clinical Diseases, Luzhou, China

**Keywords:** HBV-LC, CHB, diagnostic biomarkers, machine learning algorithms, artificial neural network, consensus clustering, immune infiltration, big data

## Abstract

**Background:** Hepatitis B-related liver cirrhosis (HBV-LC) is a common clinical disease that evolves from chronic hepatitis B (CHB). The development of cirrhosis can be suppressed by pharmacological treatment. When CHB progresses to HBV-LC, the patient’s quality of life decreases dramatically and drug therapy is ineffective. Liver transplantation is the most effective treatment, but the lack of donor required for transplantation, the high cost of the procedure and post-transplant rejection make this method unsuitable for most patients.

**Methods:** The aim of this study was to find potential diagnostic biomarkers associated with HBV-LC by bioinformatics analysis and to classify HBV-LC into specific subtypes by consensus clustering. This will provide a new perspective for early diagnosis, clinical treatment and prevention of HCC in HBV-LC patients. Two study-relevant datasets, GSE114783 and GSE84044, were retrieved from the GEO database. We screened HBV-LC for feature genes using differential analysis, weighted gene co-expression network analysis (WGCNA), and three machine learning algorithms including least absolute shrinkage and selection operator (LASSO), support vector machine recursive feature elimination (SVM-RFE), and random forest (RF) for a total of five methods. After that, we constructed an artificial neural network (ANN) model. A cohort consisting of GSE123932, GSE121248 and GSE119322 was used for external validation. To better predict the risk of HBV-LC development, we also built a nomogram model. And multiple enrichment analyses of genes and samples were performed to understand the biological processes in which they were significantly enriched. And the different subtypes of HBV-LC were analyzed using the Immune infiltration approach.

**Results:** Using the data downloaded from GEO, we developed an ANN model and nomogram based on six feature genes. And consensus clustering of HBV-LC classified them into two subtypes, C1 and C2, and it was hypothesized that patients with subtype C2 might have milder clinical symptoms by immune infiltration analysis.

**Conclusion:** The ANN model and column line graphs constructed with six feature genes showed excellent predictive power, providing a new perspective for early diagnosis and possible treatment of HBV-LC. The delineation of HBV-LC subtypes will facilitate the development of future clinical treatment of HBV-LC.

## 1 Introduction

Approximately 257 million people worldwide have CHB, and HBV-LC causes more than 700,000 deaths worldwide each year (Chen et al., 2021). 15%–20% of patients with CHB will progress to cirrhosis within 5 years (Kobashi et al., 2011). The incidence of HCC is significantly higher in patients with cirrhosis (Su et al., 2022a; Su et al., 2022b; Chen et al., 2022; Li et al., 2023a; Li et al., 2023b). Among CHB patients, the annual incidence of HCC was significantly higher in patients with cirrhosis than in non-cirrhotic patients (3.5% vs. 0.4%, *p* < 0.0001) (Do et al., 2014). However, early cirrhotic livers are in the compensated phase without any specific clinical manifestations and symptoms (Jeong et al., 2012). Patients with decompensated cirrhosis have a poor prognosis, and the 5-year survival rate for patients with untreated HBV-related decompensated cirrhosis (HBV-DeCi) is less than 15% (de Jongh et al., 1992). And only 14%–35% 5-year survival with conventional standard therapy (Wang et al., 2012; Harrison, 2015). Although most patients with HBV-DeCi can be treated with liver transplantation, the shortage of donor livers, high surgical costs and rejection make this method currently unavailable for most patients (Chan et al., 2009; Chung et al., 2014; Su et al., 2023), resulting in effective treatment is not available to many patients. Therefore, it is particularly important to improve the prognosis of HBV-LC patients by early prevention and diagnosis of the development of cirrhosis in CHB patients. Liver biopsy is currently the “gold standard” for the diagnosis of cirrhosis, but it has disadvantages such as invasiveness, complications and poor patient compliance (Spinzi et al., 2001). Various imaging tests are non-invasive and convenient (Lurie et al., 2015), but have low sensitivity and specificity for the diagnosis of early cirrhosis. Serologic tests have diagnostic value for many different types of cirrhosis (Stasi and Milani, 2017), but HBV-LC patients may show different pathologic features. Therefore, the search for new diagnostic biomarkers is crucial to improve the early diagnosis of HBV-LC patients.

With the development of bioinformatics and high-throughput sequencing technologies, mechanisms and diagnostically relevant biomarkers for a variety of liver diseases have been identified (Jin et al., 2022; Chi et al., 2023a). Although there are many studies focusing on liver diseases, studies on HBV-LC are rare. Therefore, this study aimed to identify new diagnostic biomarkers to improve the diagnosis of HBV-LC patients and elucidate the underlying molecular mechanisms of its formation. Subtyping of HBV-LC and exploring the differences between different subtypes by immunological analysis will provide new perspectives for the future treatment of HBV-LC and prevention of hepatocellular carcinoma.

In this study, we obtained the gene expression matrix file of CHB and HBV-LC through the GEO database. We screened six feature genes associated with HBV-LC formation by differential analysis of genes, WGCNA and three machine learning algorithms. Differential analysis of the feature genes was performed to explore their differential expression in CHB versus HBV-LC. Single gene GSEA analysis was performed to explore the molecular mechanism of each signature gene associated with HBV-LC formation. Functional analysis of single-sample gene set enrichment analysis (ssGSEA) hallmark gene set was performed to explore the expression differences between CHB and HBV-LC in different functions, and the correlation between the feature genes and different functions. To further study HBV-LC, we typed HBV-LC according to the feature genes in terms of molecular mechanisms and performed immune infiltration analysis for different subtypes. To provide new ideas for the diagnosis and treatment of HBV-LC. The goal of our study is to diagnose and analyze the formation of HBV-LC more comprehensively through genetic data.

## 2 Materials and methods

### 2.1 Acquisition and processing of gene expression data

The expression matrix files of all datasets GSE114783 (10CHB, 10HBV-LC), GSE84044 (43CHB, 10HBV-LC), GSE123932 (6HBV-LC), GSE121248 (6CHB) and GSE119322 (37CHB) used in this study were obtained from the NCBI Gene Expression Omnibus database (GEO, https://www.ncbi.nlm.nih.gov/geo/). We combined the samples studied in the two datasets GSE114783 and GSE84044, and then removed the combined batch effects as the training set. The amalgamation of samples from the three datasets, namely, GSE123932, GSE121248, and GSE119322, culminated in the formation of a unified cohort. Subsequent to this amalgamation, diligent measures were employed to mitigate any batch effects inherent in the combined dataset. This meticulously curated amalgamated dataset was then earmarked for deployment as the validation set in the study.

### 2.2 Screening and enrichment analysis of differential genes

Differential gene expression analysis was performed on the training set using the R package “Limma” to identify differential genes (DEGs) (|log2FC|>1, FDR<0.05) (Zhao et al., 2022a; Zhao et al., 2022b; Li et al., 2022). We also used the R package “pheatmap” and “ggplot2″ to create heat maps and volcano maps to visualize the DEGs (Song et al., 2022a; Song et al., 2022b). To investigate the enrichment levels of DEGs in different functional pathways, we performed gene set enrichment analysis (GSEA) on DEGs using the R package “clusterProfiler” (Zhong et al., 2022; Huang et al., 2023a; Zhao et al., 2023), and selected the five highest and lowest enriched functional pathways for visualization.

### 2.3 Construction of gene co-expression networks

The WGCNA method is useful for gene expression studies. The R package “WGCNA” is used to develop and modularize gene co-expression networks (Song et al., 2023; Zhang et al., 2023; Song et al., 2023). The samples are clustered to detect any significant outliers or outliers. Thereafter, automated networks are used to build co-expression networks. Detection of modules is performed using hierarchical clustering and dynamic tree cut. Module membership (MM) and gene significance (GS) evaluations are used to associate modules with disease characteristics. Core co-expression modules were those with the highest module membership (MM) and *p* < 0.05. MM > 0.8 indicated high module relevance and GS > 0.2 indicated high clinical importance. Modules with the highest correlation with patient groups were selected to provide disease-associated co-expressed genes for further studies.

### 2.4 Identify candidate genes and perform functional enrichment analysis

The intersection of DEGs with WGCNA disease correlations was taken as a candidate gene using a Venn diagram. To evaluate the candidate genes for disease-related diseases, Disease Ontology (DO) analysis was performed using the R package “DOSE”. To further determine the potential biological functions and signaling pathways of the candidate genes, functional enrichment analysis was applied to evaluate the candidate genes. Gene Ontology (GO) and Kyoto Encyclopedia of Genes and Genomes (KEGG) analyses were performed using the R package “clusterProfiler” (Zhang et al., 2022; Shen et al., 2022; Huang et al., 2023; Zhang et al., 2023).

### 2.5 Machine learning screens for diagnostic biomarkers

Three machine learning algorithms, SVM-RFE, RF and LASSO logistic regression (Chi et al., 2023b; Ren et al., 2023), were chosen to jointly screen diagnostic biomarkers. The SVM-RFE algorithm was performed using the R package “caret” and “e1071” to train different types of feature subsets and calculate the points with the lowest cross-validation error (Jin et al., 2021a). The number of genes with the minimum error is the number of diagnostic biomarkers screened by SVM-RFE (Chi et al., 2023c). The RF algorithm was performed using the R package “randomForest” to rank the relative importance of the candidate genes, and those with relative importance >1.25 were selected as the diagnostic biomarkers screened by the RF algorithm. After that, we used the R package “glmnet” to perform LASSO logistic regression and adjusted the optimal penalty parameter λ for 10-fold cross-validation to obtain the diagnostic biomarkers screened by LASSO logistic regression (Zhang et al., 2023). Finally, we obtained six disease-related feature genes by taking the intersection of the three machine learning screened feature genes.

### 2.6 Construction and validation of diagnostic models

We used the R packages “neuralnet” and “dplyr” to construct an ANN model with a nomogram model (Jin et al., 2022). The expression data of the screened feature genes were extracted. The median expression value of each characteristic gene in all samples was used as the criterion to score each feature gene in each sample with Gene Score. The Gene Score for each gene in the training set was obtained by using the following rules: 1 for upregulated genes above the median and 0 for downregulated genes above the median, and 0 for downregulated genes above the median and 1 for downregulated genes below the median. The neural network model was constructed using the R package “neuralnet”, which consists of an input layer, a hidden layer and an output layer, and the output layer was obtained by multiplying the gene score and the gene weight (formula: Neural HBV-LC = *Σ*(Gene Score*Gene Weight)). The performance of the model was assessed by plotting the subject worker curve (ROC) using the R package “PROC” and was specified by the area under the ROC curve (AUC) (Jin et al., 2021b). To demonstrate the generalizability of the model, the validation set gene expression matrix was transformed into its corresponding Gene Score using the same method, and the AUC of the validation set was obtained to assess whether the generalizability of the model was good. In addition, a nomogram was constructed using the R package “Rms” to predict the conversion of CHB patients to HBV-LC (Dong et al., 2023). The expression of each characteristic gene was obtained as a score for the relevant gene, and the total score was obtained by summing all the scores to predict the risk of disease based on the total score. The predictive power of the nomogram model was assessed by calibration curves. The clinical utility value of the model is evaluated by decision curve analysis (DCA).

### 2.7 Functional analysis of ssGSEA hallmark gene set

The GSEA website (https://www.gsea-msigdb.org/gsea/index.jsp) provides the required hallmark gene set data for this study. The functional scores of the corresponding gene sets were obtained for each sample using the R packages “GSVA” and “GSEABase” (Huang et al., 2023c). The differences in expression of biological functions between the disease and control groups were then compared. To analyze the correlation between feature genes and biological functions, a functional correlation test was performed for each feature gene.

### 2.8 Consensus clustering of diseases

The disease under study can be classified into different subtypes by consensus clustering. The expression matrices of HBV-LC patients in the training and validation sets were combined and the characteristic gene expressions of all disease samples were extracted as the original expression matrix for consensus clustering. HBV-LC patients were classified into different subtypes using the R package “ConsensusClusterPlus”. The results were repeatedly run 1,000 times to verify the accuracy and reproducibility of the consensus clustering. The optimal number of clusters was determined by the relative changes in the consensus matrix plot and the consensus cumulative distribution function (CDF) plot, as well as the trajectory plot. To verify the clustering effect, PCA plots were drawn using the R package “ggplot2” to observe whether the samples of different subtypes were separated on the scatter plot. The heat map and box line plot between genes and different subtypes were also used to observe the expression and expression differences between different subtypes for each feature gene.

### 2.9 Gene set variance analysis (GSVA)

GSVA analysis was performed on different subtypes of HBV-LC by using the gene set selected from MSigDB as the reference set and the screened feature genes as the sample gene set. The GSVA score of each gene set was obtained using the R package “GSVA” (Zhang et al., 2022). The scores indicate the absolute enrichment of the gene sets to investigate whether they are enriched in different functional pathways among different subtypes.

### 2.10 Immune infiltration analysis

CIBERSORT is widely used for the estimation of immune cell composition based on the expression matrix of genes. With the CIBERSORT algorithm we quantified the proportion of 22 immune cell infiltrations between patients with different subtypes of HBV-LC and normal subjects (Cui et al., 2022), showing the proportion of immune cells in different subtypes of samples in the form of bar charts. The correlation between the 22 immune cell types was analyzed using the R package “corrplot” and presented as a heat map. The expression differences of 22 immune cells and 10 immune functions were compared between different subtypes by box plot (Wang et al., 2023).

### 2.11 Statistical analysis

All analyses were performed statistically using R software version 4.2.2. The Wilcoxon rank sum test was used to compare the proportion of immune function and immune cell infiltration for different subtypes of HBV-LC. Correlation coefficients were tested using spearman correlation analysis. A *p*-value < 0.05 or false discovery rate (FDR) < 0.05 was considered statistically significant in all statistical findings of this study.

## 3 Results

### 3.1 Identification of DEGs

**FIGURE 1:**
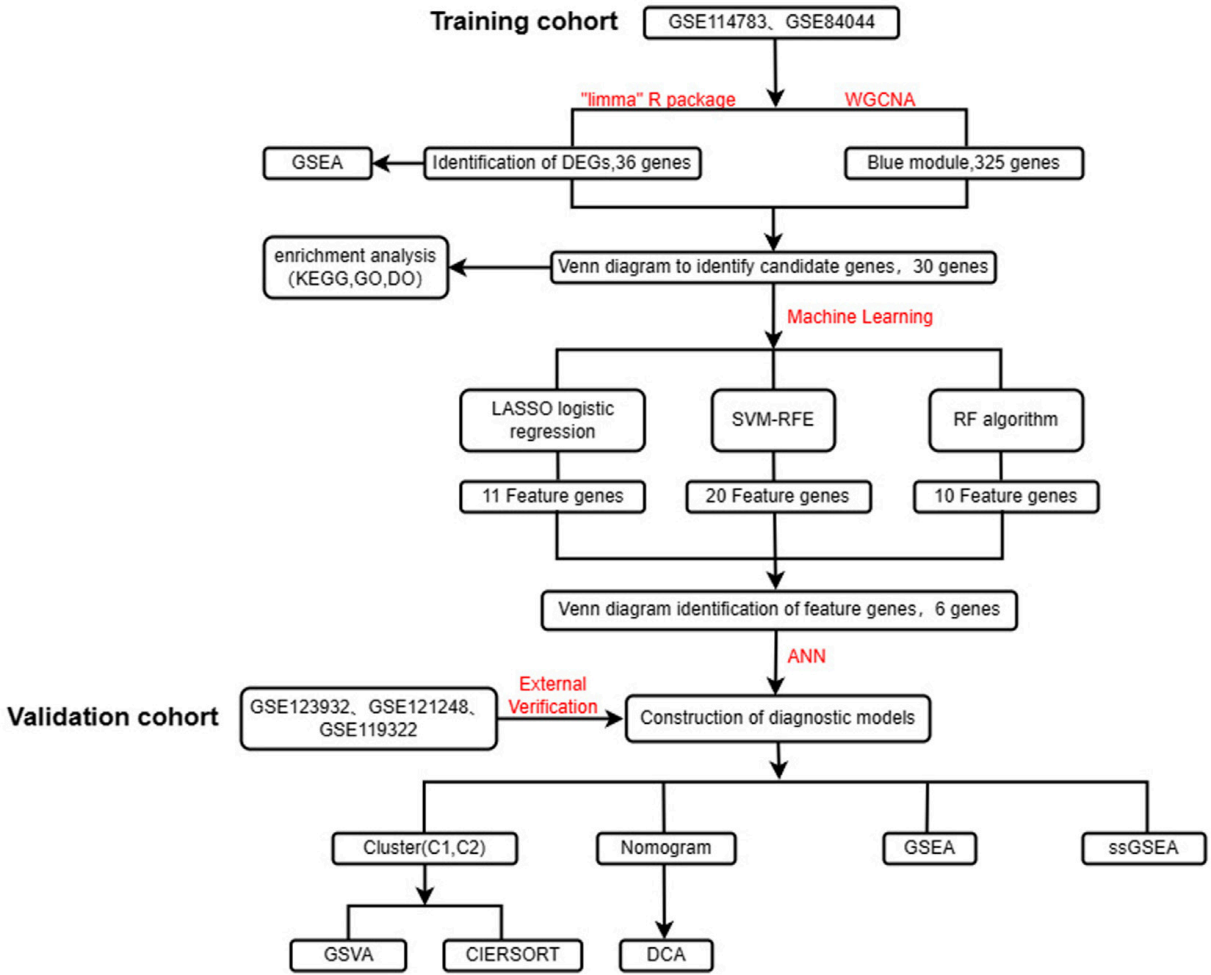
Flow chart of the design of this study.

**FIGURE 2:**
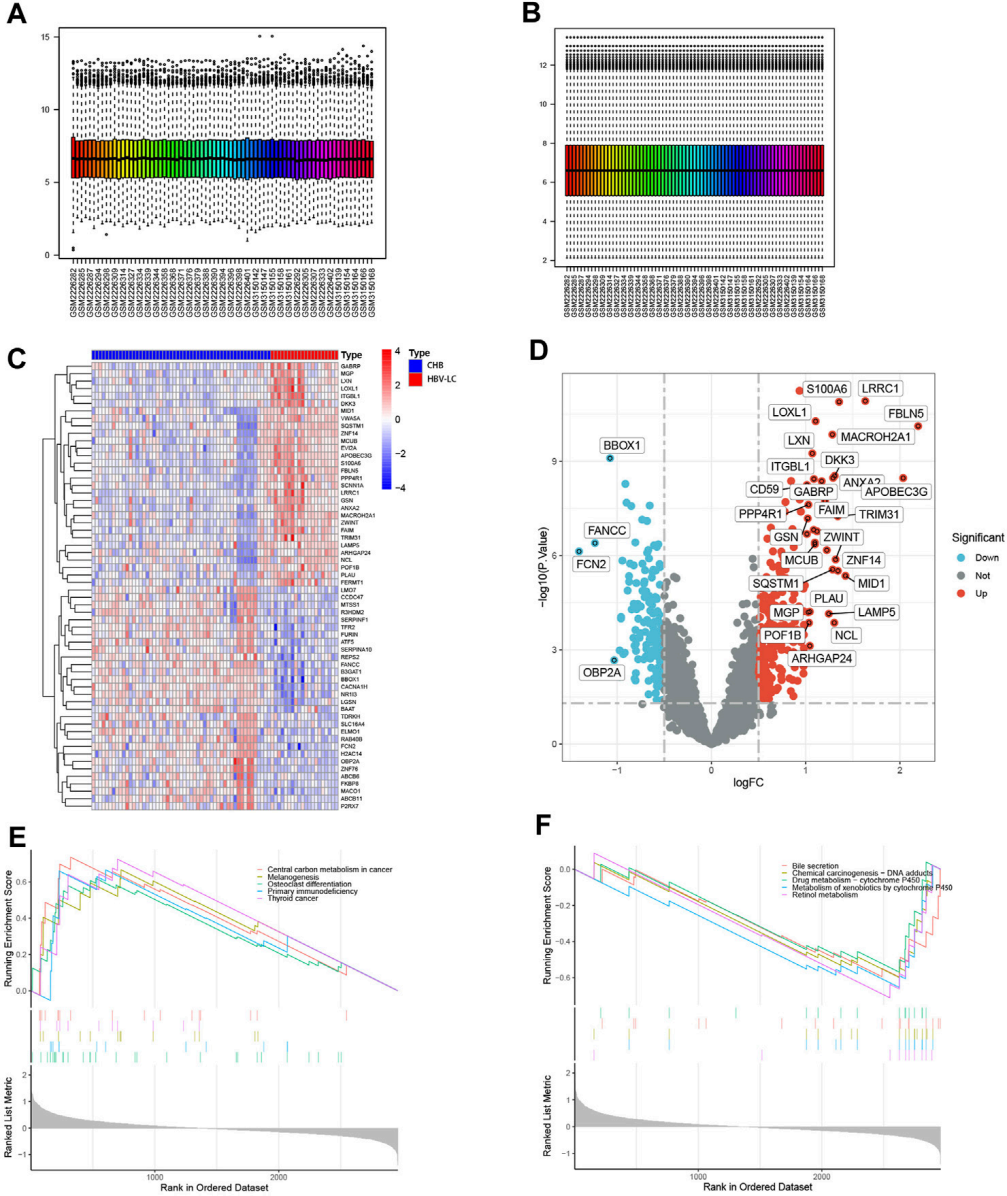
Identification of DEGs between CHB and HBV-LC samples with analysis. **(A,B)** Batch correction of the combined samples to remove the effect from batch effects. **(C)** Heat map of differential genes, where C represents the control group consisting of CHB patient samples and P represents the patient samples of HBV-LC for the disease group. Red represents genes with high expression in that sample and blue represents genes with low expression in that sample. The distribution of differential genes between the control and disease groups can be seen by observing the correlation between heat map genes and samples. **(D)** Volcano plots of differential genes. With log2FC = 0 and *p* < 0.05 as the criterion, greater than 0 is upregulated and less than 0 is downregulated. We selected genes satisfying |log2FC|>1, *p* < 0.05 as genes with significant differences and showed their names on the volcano plots. **(E,F)** GSEA analysis of differential genes, and the top five functional pathways with up- and downregulated expression were selected for display.

Figure 1 shows the flow chart of this study. To identify DEGs between the CHB and HBV-LC groups, the expression matrices of CHB and HBV-LC samples in the two datasets GSE114783 and GSE84044 were merged in this study, and the merged data were batch corrected to remove the batch effect from the merger (Figures 2A, B). A total of 36 genes (|log2FC|>1, FDR<0.05) were significantly differentially expressed in the combined data. Among them, 4 genes were downregulated and 32 genes were upregulated (Figures 2C, D). In addition, to understand the enrichment level of DEGs in different functional pathways, we performed GSEA analysis on the combined datasets, and the top 5 most significantly enriched pathways were shown (Figures 2E, F). The results showed that the pathway with significant enrichment of DEGs was Central carbon metabolism in cancer, indicating that DEGs may affect the conversion of CHB patients to HBV-LC or even HCC. And these DEGs were downregulated in the expression of several pathways of Bile secretion, Drug metabolism-cytochrome P450, Metabolism of xenobiotics by cytochrome P450, Retinol metabolism, all associated with the metabolic function of hepatocytes. The above results suggest that these genes may be associated with functional impairment of hepatocytes.

### 3.2 WGCNA establishes gene co-expression network

**FIGURE 3:**
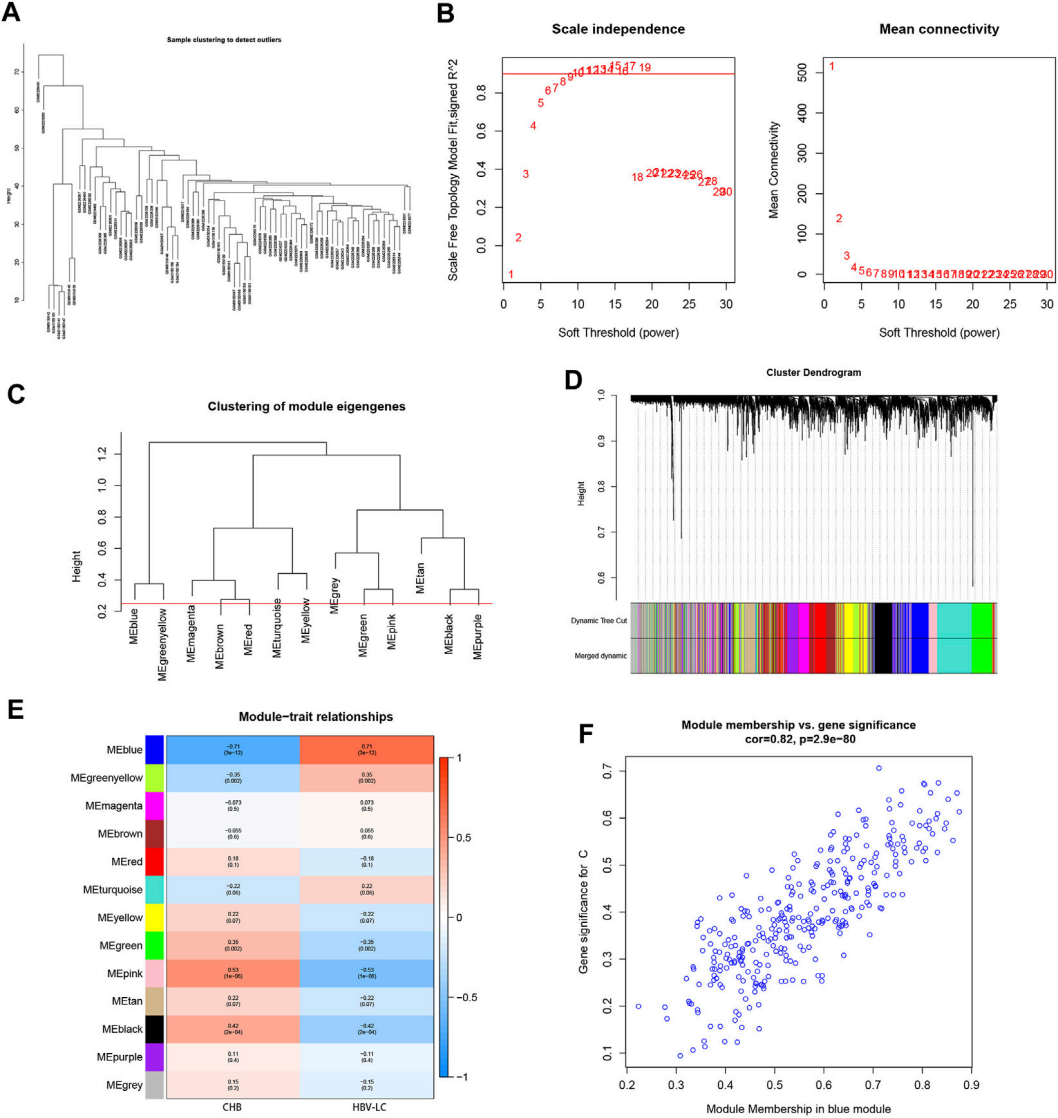
Establishment of WGCNA gene co-expression network and module selection. **(A)** The samples were clustered to construct the sample clustering dendrogram to observe whether there are outliers **(B)** The scale-free topological fit index R^^2^ was set to 0.9, when the soft threshold *β* = 9 and the average connectivity of the network was good. **(C)** The height is set to 0.25 to cut the clustered dendrograms to detect expression modules that are similar to the combination. **(D)** Sample clustering trees with their original and combined modules were displayed to observe the clustering effect. **(E)** Heat map of correlations between modules and disease features. Red represents positive correlation and blue represents negative correlation. The values among the brackets are *p*-values to test whether the modules are statistically significant. The values above the brackets represent the magnitude of correlation between modules and clinical features. **(F)** Scatterplot of the correlation between the MEblue module, which has the strongest correlation with HBV-LC, and the disease-related genes, with an overall trend of positive correlation.

To screen for co-expressed genes associated with HBV-LC, weighted gene co-expression network analysis (WGCNA) was performed on the merged dataset. A threshold was set to cluster all samples to observe whether there were outliers or abnormal values (Figure 3A), and obviously abnormal samples were removed. The co-expression network was constructed, and the average connectivity of the network was good when the scale-free topological fit index R^2 > 0.9, so we set a soft threshold *β* = 9 (Figure 3B). The clustering height was set to 0.25, and the modules with strong correlation were merged (Figure 3C), and in this way 13 modules were identified for the subsequent study. The sample clustering tree was displayed with its original and merged modules (Figure 3D), and the results of the merged and pre-merged modules with strong associations could be observed. The relationship between modules and clinical symptoms was investigated by correlation between ME values and disease characteristic. To study the co-expressed genes associated with HBV-LC, the 13 co-expressed modules in the heat map were observed. There was a negative correlation between the blue module and the control group (*r* = −0.71, *p* = 3e-12) and a positive correlation with the patient group (*r* = 0.71, *p* = 3e-12) (Figure 3E), so the most significant correlation between the blue module and HBV-LC was found for 325 genes. After identifying clinically significant modules, a review of the patient group MM versus GS scatter plot showed that the blue module was highly correlated with HBV-LC (Figure 3F). All genes of this module will be used for further studies.

### 3.3 Identification of candidate genes and functional enrichment analysis

**FIGURE 4:**
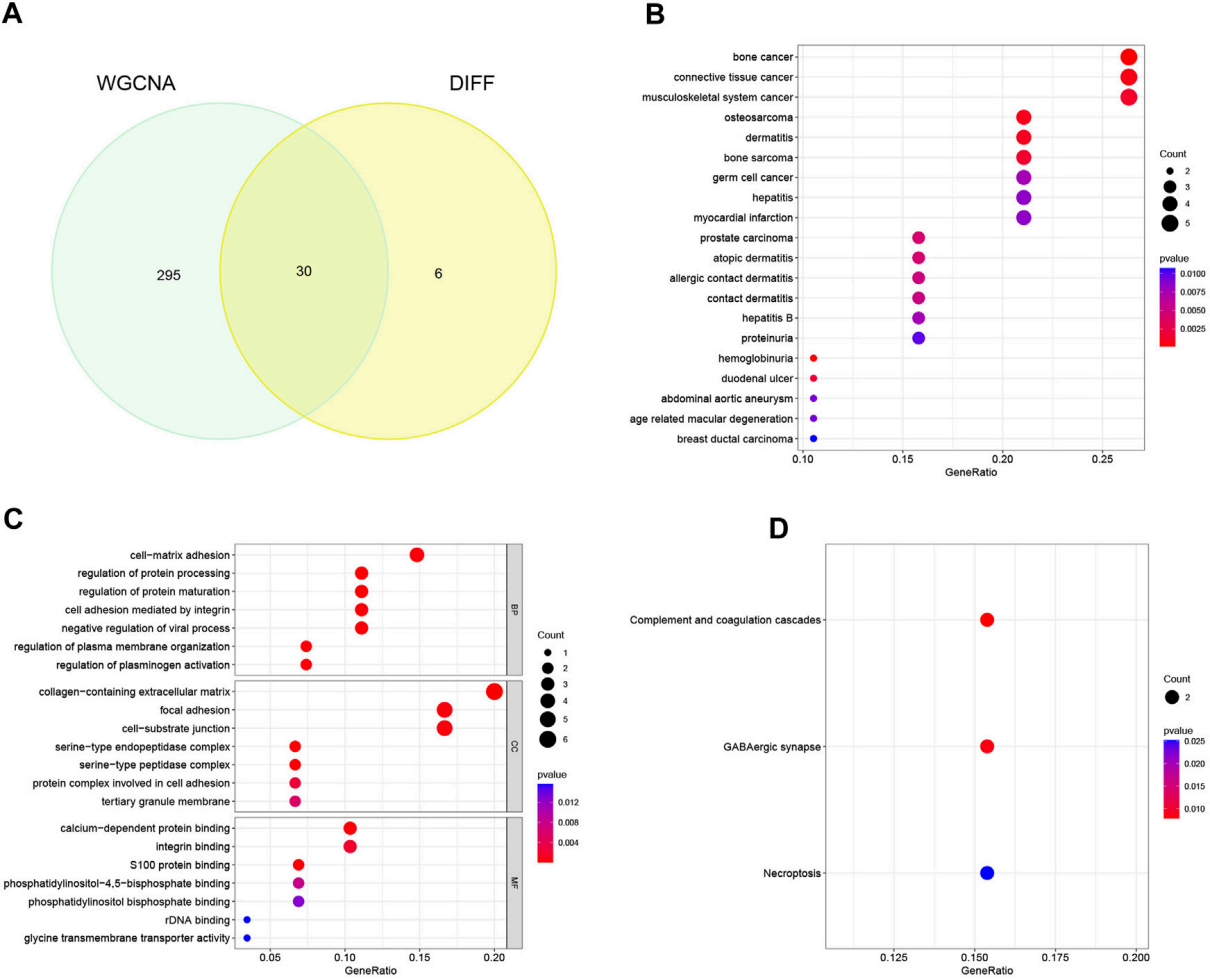
Identification of candidate genes and analysis of functional enrichment. **(A)** Venn diagram of disease-associated co-expression module genes screened by WGCNA with differential genes, intersecting genes as candidate genes. **(B)** Bubble plots of candidate genes DO analysis. **(C)** Bubble plot of candidate genes GO analysis. **(D)** Bubble plot of candidate genes KEGG analysis.

Through meticulous execution of a Venn diagram analysis, we successfully identified and delineated a cohort of 30 candidate genes that exhibited a pronounced and statistically significant correlation between DEGs and the context of HBV-LC (Figure 4A). Subsequently, a comprehensive Disease Ontology (DO) enrichment analysis was meticulously undertaken to unravel the intricate implication of these candidate genes within the disease landscape (Figure 4B). The discerning outcomes of this analysis not only underscored but also substantiated the substantial enrichment of these candidate genes within the domain of hepatocellular afflictions, specifically hepatitis and hepatitis B. To illuminate the biological processes and pathways intricately linked to these select genes, a meticulous exploration involving Gene Ontology (GO) and Kyoto Encyclopedia of Genes and Genomes (KEGG) enrichment analyses was meticulously undertaken. In the realm of GO enrichment analysis, it was intriguingly revealed that the candidate genes actively partake in a spectrum of pivotal biological processes (BP), encompassing fundamental aspects such as cell-matrix adhesion and the constraining of viral processes. Furthermore, at the level of molecular function (MF), these candidate genes intricately engage in functionalities intrinsic to the collagen-containing extracellular matrix, as well as the pivotal phenomenon of focal adhesion, among other pivotal roles. Delving into the intricacies of cellular components (CC), it is noteworthy that these genes find their habitat within crucial domains such as calcium-dependent protein binding and integrin binding, contributing substantively to cellular architecture and function (Figure 4C). To widen the scope, the KEGG enrichment analysis astutely divulged the predominant involvement of the candidate genes in pathways of paramount significance, particularly those associated with tissue injury and cellular demise. Among these pathways are the intricate complement and coagulation cascades, along with the consequential Necroptosis pathway (Figure 4D).

### 3.4 Machine learning screens for diagnostic biomarkers

**FIGURE 5:**
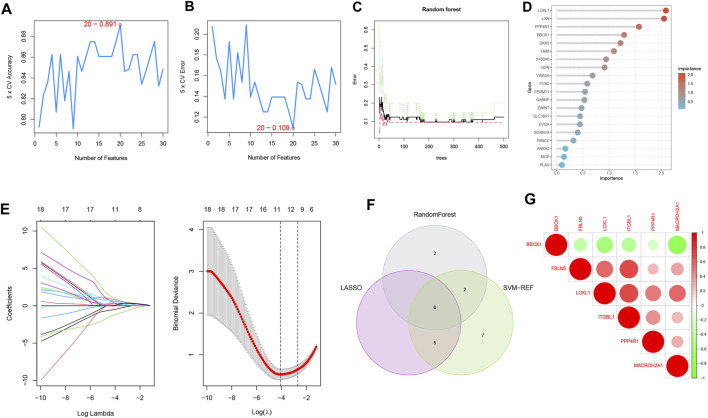
Machine learning screening of diagnostic biomarkers. **(A,B)** Support vector machine recursive feature elimination (SVM-RFE) algorithm for screening biometric genes, with the number of points with the lowest accuracy and error rate as feature genes. **(C)** Number of random forest (RF) algorithm error rate and classification trees. **(D)** Relative importance of the top 20 genes. **(E)** Least absolute shrinkage and selection operator (LASSO) logistic regression algorithm for screening feature genes. The distribution of LASSO coefficients for the 11 genes that initially met the diagnostic criteria versus the amount of genes determined by sample binomial deviation. **(F)** Venn diagram of three machine learning algorithm feature genes taken as diagnostic biomarkers. **(G)** Heat map of correlation analysis among the signature genes, the size of the circles represents the size of correlation between genes, red represents positive correlation and green represents negative correlation.

To select reliable diagnostic biomarkers from candidate genes, we used three machine learning algorithms, including SVM-RFE, RF, and LASSO logistic regression. The SVM-RFE algorithm was calculated to select the top 20 genes with the highest accuracy (Figure 5A) and the lowest error rate (Figure 5B). The RF algorithm combined the error rate with the number of classification trees (Figure 5C). The relative importance of candidate genes was ranked, and the 10 candidate genes with relative importance >1.25 were selected as diagnostic biomarkers for RF algorithm screening (Figure 5D). 11 genes were screened from the statistically significant univariate variables by LASSO regression analysis (Figure 5E). The intersection of the three machine learning algorithms was obtained by Wayne diagram, and six diagnostic biomarkers were screened as PPP4R1, BBOX1, LOXL1, MACROH2A1, ITGBL1, and FBLN5 (Figure 5F). The screened diagnostic biomarkers were used as the feature genes of HBV-LC. Correlation tests between genes were performed for the feature genes (Figure 5G), and the results showed that BBOX1 was negatively correlated with the remaining five genes, indicating a significant functional difference between BBOX1 and the remaining five genes. And five genes, LOXL1, MACROH2A1, ITGBL1, FBLN5 and PPP4R1, were all positively correlated with each other, indicating a significant functional similarity between these five genes.

### 3.5 Construction of diagnostic models, validation and construction of column line graphs

**FIGURE 6:**
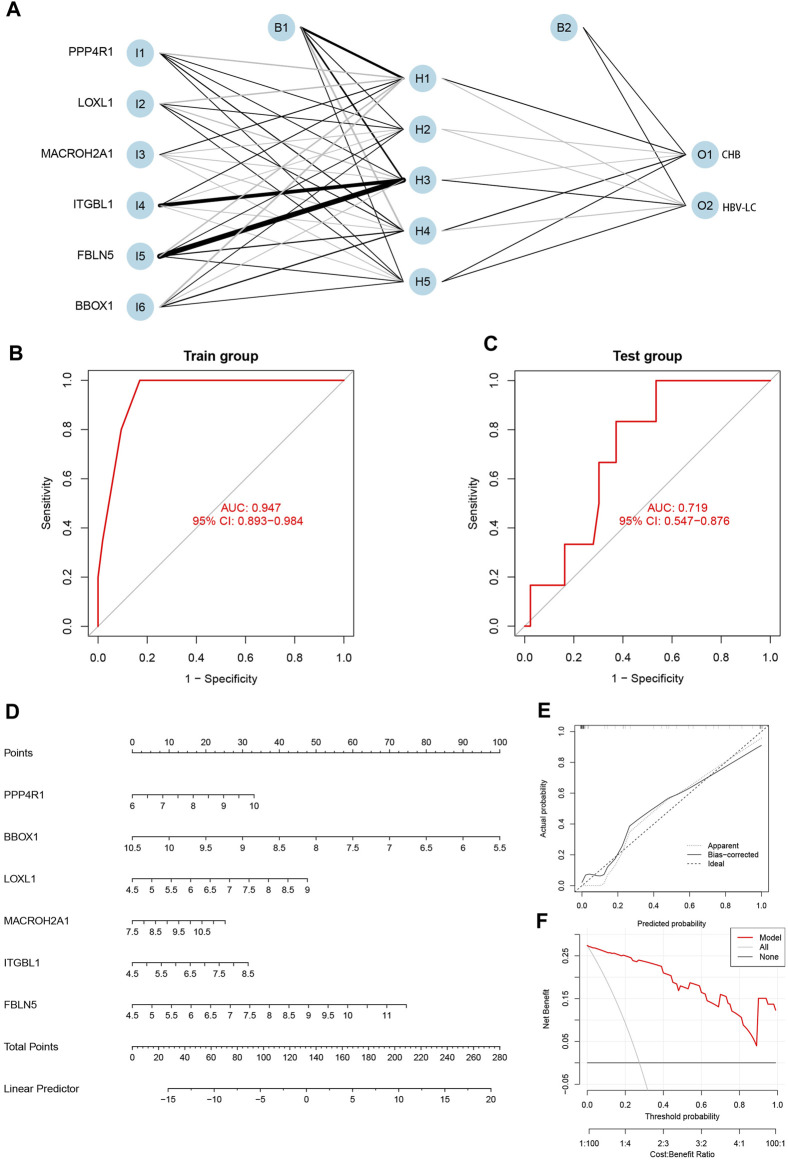
Construction and validation of the diagnostic model. **(A)** Construction of the artificial neural network (ANN) model, which contains the input layer, hidden layer and output layer. **(B)** Training set receiver operating characteristic curve (ROC), the size of the area under the curve (AUC) demonstrates the feasibility of the model. **(C)** Validation set ROC, the size of AUC can indicate the applicability of the model. **(D)** Diagnostic line graph of HBV-LC feature genes, each gene corresponds to a score, and the scores of all genes are summed to obtain the total score. **(E)** Calibration curve to assess the predictive performance of the diagnostic line graph. **(F)** Decision curve analysis (DCA), which compares the clinical benefit of the diagnostic line graph model with other diagnostic indicators.

The integration of the expression matrix containing the meticulously screened six genes with the amalgamated expression matrix provided a robust foundation for analysis. In order to ascertain the significance of these pivotal genes, a “gene score” dichotomizing between 0 and 1 was ascribed based on the median expression value observed across the entire spectrum of samples. Subsequent to this, the computation of feature gene weights facilitated the construction of an innovative Artificial Neural Network (ANN) model (as expressed in the equation: Neural HBV-LC = *Σ*(Gene Score*Gene Weight)). The architecture of the ANN model comprised a well-defined input layer, a discreet hidden layer, and a discerning output layer (Figure 6A). The efficacy of the ANN model was underscored through rigorous training with a designated dataset, culminating in an impressive area under the receiver operating characteristic curve (ROC AUC) of 0.947—a testament to its prowess in prognosticating HBV-LC (Figure 6B). Furthermore, the application of the identified feature genes along with their corresponding gene scores to an independent validation dataset yielded an AUC value of 0.719, thereby substantiating the model’s robustness and real-world applicability (Figure 6C). Elevating the clinical utility of HBV-LC risk assessment, we meticulously constructed a column line graph model for precise HBV-LC diagnosis, seamlessly employing the six pivotal genes in conjunction with the R package “Rms” (Figure 6D). The precision of the model was reiterated by the alignment of calibration curves, demonstrating minimal disparities between predicted and actual HBV-LC risk levels (Figure 6E). Crucially, the discernment offered by Decision Curve Analysis (DCA) emphasized the superior diagnostic utility of the “model” curve in comparison to the “ALL” curve, discernibly enhancing clinical diagnostic precision within the 0–1 threshold range (Figure 6F).

### 3.6 Differential expression of feature genes

**FIGURE 7:**
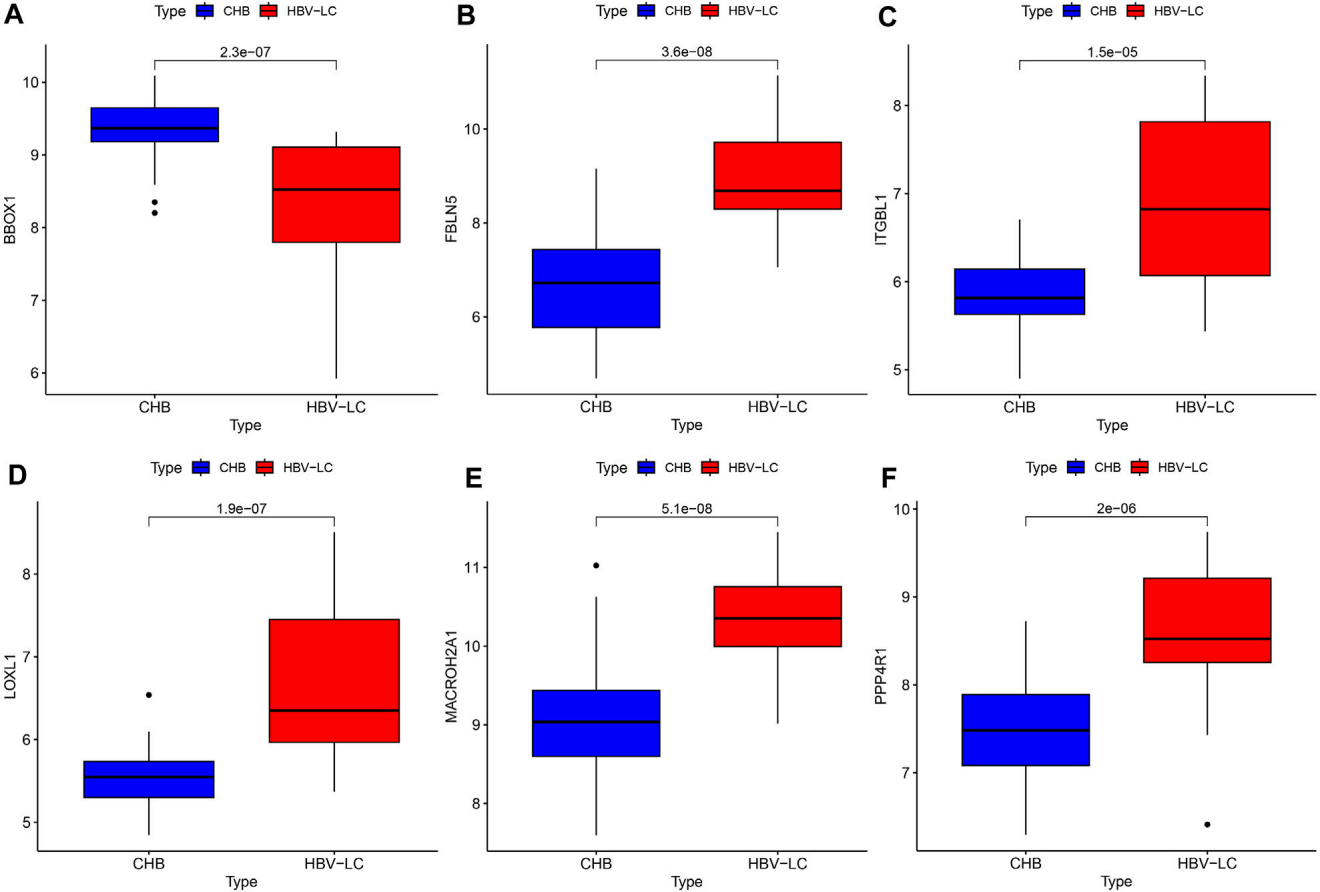
Feature gene expression differences. **(A–F)** Box line plots of expression differences between patient and control groups for each feature gene, compared as the mean of gene expression box line plots, with the numbers on them representing *p* values.

To explore the difference in expression of the screened feature genes in the patient group versus the control group, we plotted the box plots of gene expression differences. Under the condition of meeting statistical significance, the results showed that the expression of BBOX1 was lower in the disease group than in the control group (Figure 7A). While the expression of the remaining five genes, FBLN5, ITGBL1, LOXL1, MACROH2A1 and PPP4R1, was higher in the patient group than in the control group (Figures 7B–F).

### 3.7 Single-gene GSEA analysis of feature genes and functional analysis of ssGSEA hallmark gene set

**FIGURE 8:**
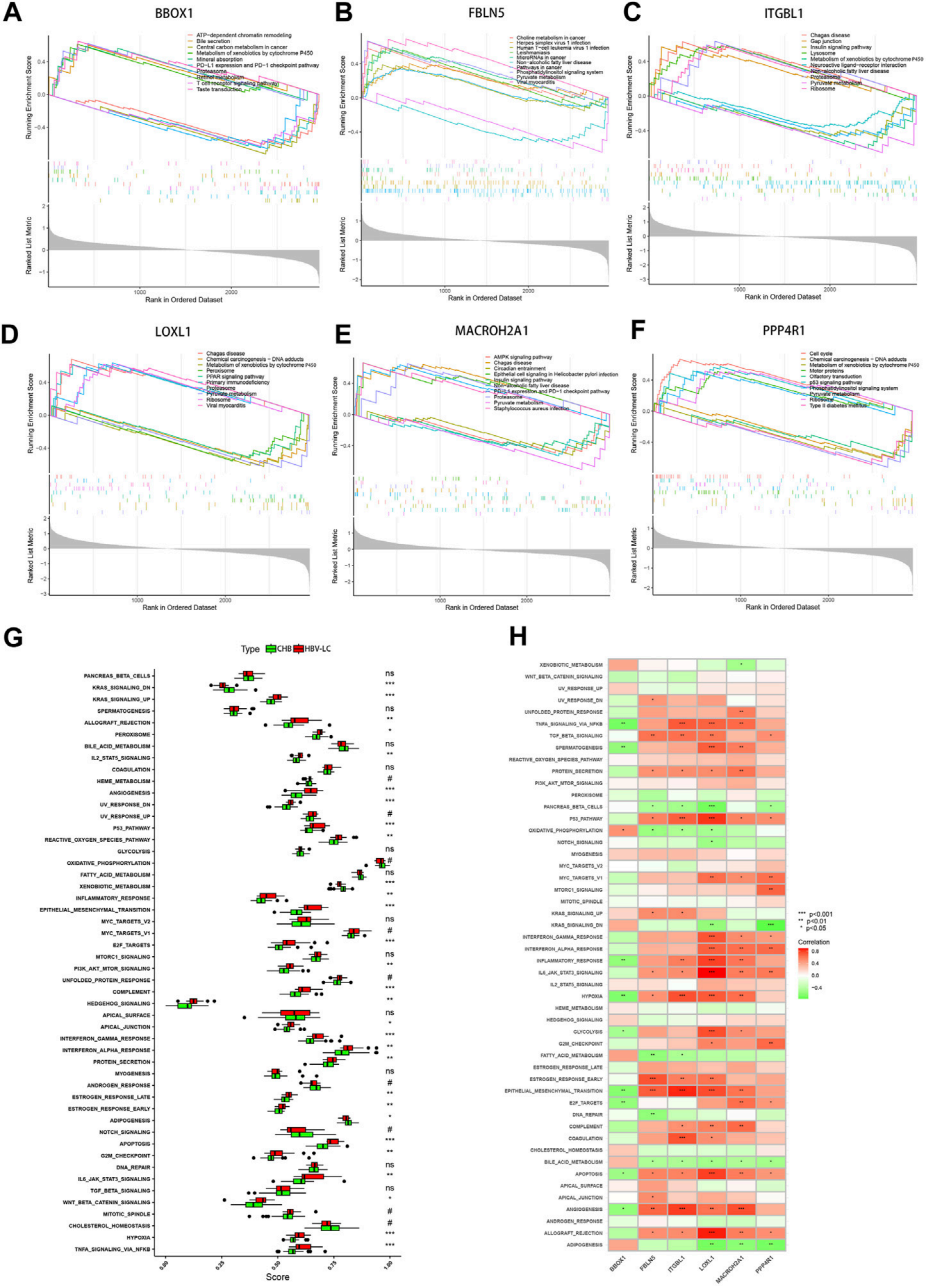
GSEA and ssGSEA functional enrichment analysis of the feature genes. **(A–F)** Single-gene GSEA analysis of the feature genes, showing the top 5 functional pathways upregulated versus downregulated for each gene. **(G)** Box line plot of differential expression of functional ssGSEA scores of signature gene sets between patient groups and control groups. **(H)** Correlation between feature genes and function, red represents positive correlation, green represents negative correlation. *p < 0.05, **p < 0.01, ***p < 0.001. ns, no significance.

To elucidate the intricate molecular underpinnings governing the transition from CHB to hepatitis HBV-LC, we embarked upon a comprehensive endeavor employing single gene GSEA on the six pivotal genes that emerged from our screening efforts. This exploration delved into the gene expression patterns within diverse biological processes (Figures 8A–F). Notably, within this gamut, the gene BBOX1 garnered attention for its heightened expression in the pathway associated with bile secretion, concurrently exhibiting subdued expression in the context of disease groups. Evidently, BBOX1’s downregulated expression could conceivably attenuate bile secretion in damaged hepatocytes. Intriguingly, FBLN5 and PPP4R1 exhibited pronounced activity within the Phosphatidylinositol signaling system, hinting at their potential involvement in orchestrating the conversion from CHB to HBV-LC through the modulation of phosphatidylinositol-mediated signaling cascades. Meanwhile, ITGBL1, LOXL1, and MACROH2A1, encompassing three genes, exhibited heightened expression within the Proteasome pathway, while PPP4R1 emerged as a prominent contender within the p53 signaling pathway. This conveys that these four genes might intricately govern the transition by regulating cell cycle dynamics. Pertinently, the elevated expression of ITGBL1, LOXL1, and PPP4R1 also exhibited correlations with the Ribosome pathway, suggesting their potential sway over ribosomal functionality. Extending our investigation, we directed our focus towards assessing hallmark gene sets’ functional analyses between CHB and HBV-LC through ssGSEA. Strikingly, upon meticulous curation, these analyses revealed a consistent trend wherein the expressions across various functional categories within patient groups surpassed those in the control cohort. Notwithstanding, a trio of functional disease groups - KRAS_SIGNALING_DN, XENOBIOTIC_METABOLISM, and ADIPOGENESIS - exhibited relatively diminished expression as compared to the control group (Figure 8G). Building upon this foundation, we delved into an intricate investigation of the functional correlation existing between individual genes and the hallmark gene sets. As a result, it became patently evident, after rigorous filtration of statistically insignificant functions, that these genes exhibited strikingly significant positive or negative correlations with their corresponding functions (Figure 8H). This intriguing interplay underscores the pivotal role that these feature genes assume in modulating the manifestation of these highly expressive functions, which, in turn, influences the nuanced journey of CHB transformation into HBV-LC. In summation, our comprehensive analyses paint a vivid portrait of the molecular choreography orchestrating the conversion, underscoring the intricate network of interrelated processes that govern this transition.

### 3.8 Consensus clustering of HBV-LC by feature genes

**FIGURE 9:**
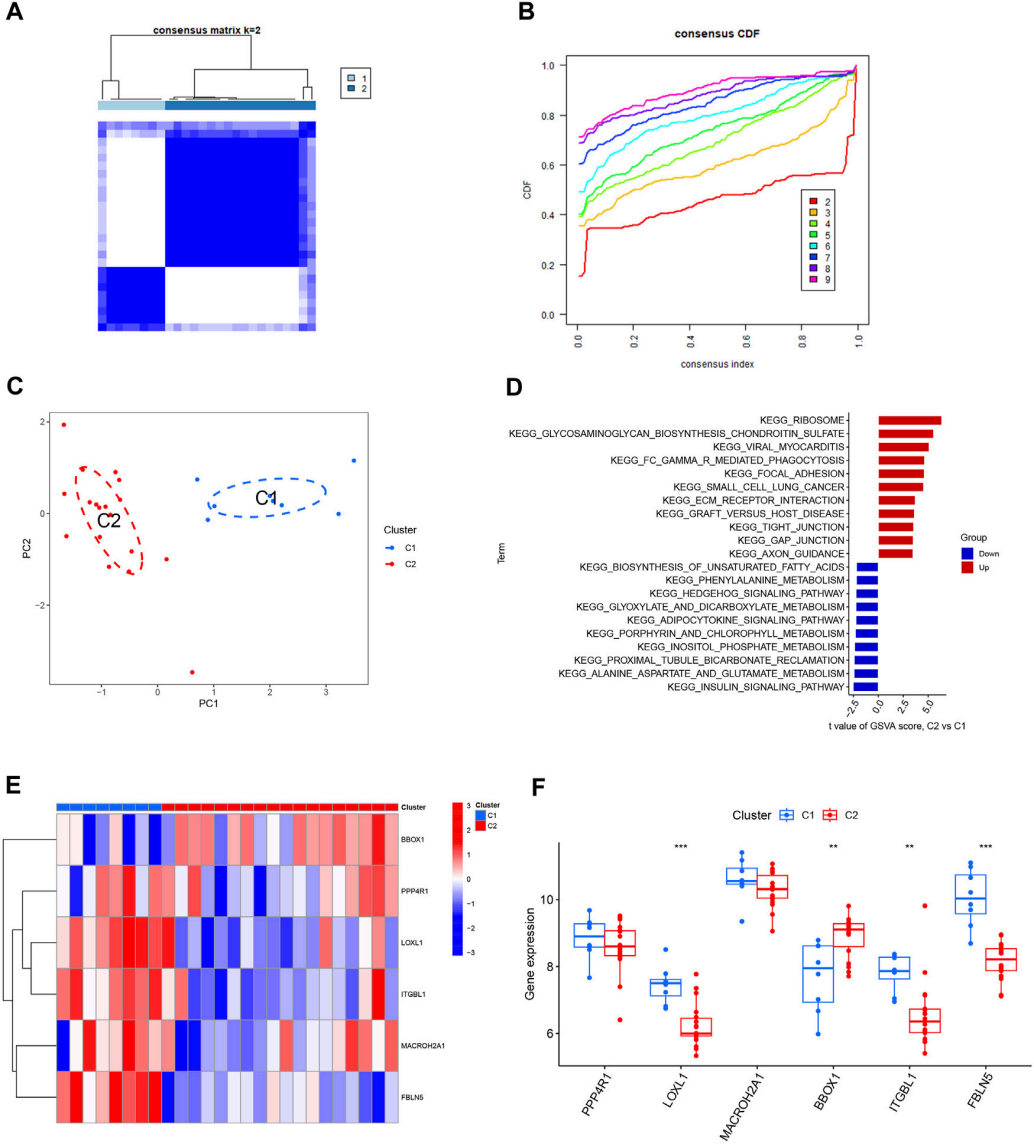
Typing and validation of HBV-LC based on feature genes. **(A)** Consensus matrix heat map of disease typing, by observing whether the blank area between different subtypes is clean to initially determine the typing effect, *k* = 2. **(B)** Consensus CDF map classified as k = 2-9, by observing the dynamic change of consistency index with the change of CDF value for different subtype numbers, *k* = 2 was selected. **(C)** PCA map of disease samples, the feature genes effectively classified hepatitis B-related cirrhotic patients into two subtypes (C1 and C2). **(D)** GSVA analysis between different subtypes of HBV-LC. **(E)** Heat map showing the expression of characteristic genes between two subtypes of HBV-LC, C1 and C2. **(F)** Box line plot of gene expression differences between two subtypes of C1 and C2. *p < 0.05, **p < 0.01, ***p < 0.001.

The HBV-LC samples in the training and validation sets were combined to obtain the expression matrix required for typing. The subtyping of HBV-LC samples was performed by consensus clustering based on the six feature gene expression profiles screened. By the consensus matrix plot with CDF curve, we selected the optimal number of typing *k* = 2 (Figures 9A, B) and classified the samples into two subtypes C1 and C2. Validation was performed by PCA plots, and the results showed that there was distinguishable between the two subtypes on the scatter plot (Figure 9C). GSVA analysis was performed on both C1 and C2 subtypes, and the results showed that the Ribosome pathway plays an important role in the division of the two subtypes. It indicates that the signature genes can be involved in HBV-LC typing by regulating the ribosome function (Figure 9D). Excluding the results without statistical significance, LOXL1, ITGBL1 and FBLN5 were expressed upregulated in C1 subtype C2 subtype expression was downregulated. While BBOX1 was downregulated in C1 subtype expression C2 subtype expression was upregulated (Figures 9E, F). Thus by the expression of these feature genes we can classify HBV-LC into two subtypes.

### 3.9 Immune infiltration analysis of two subtypes of HBV-LC

**FIGURE 10:**
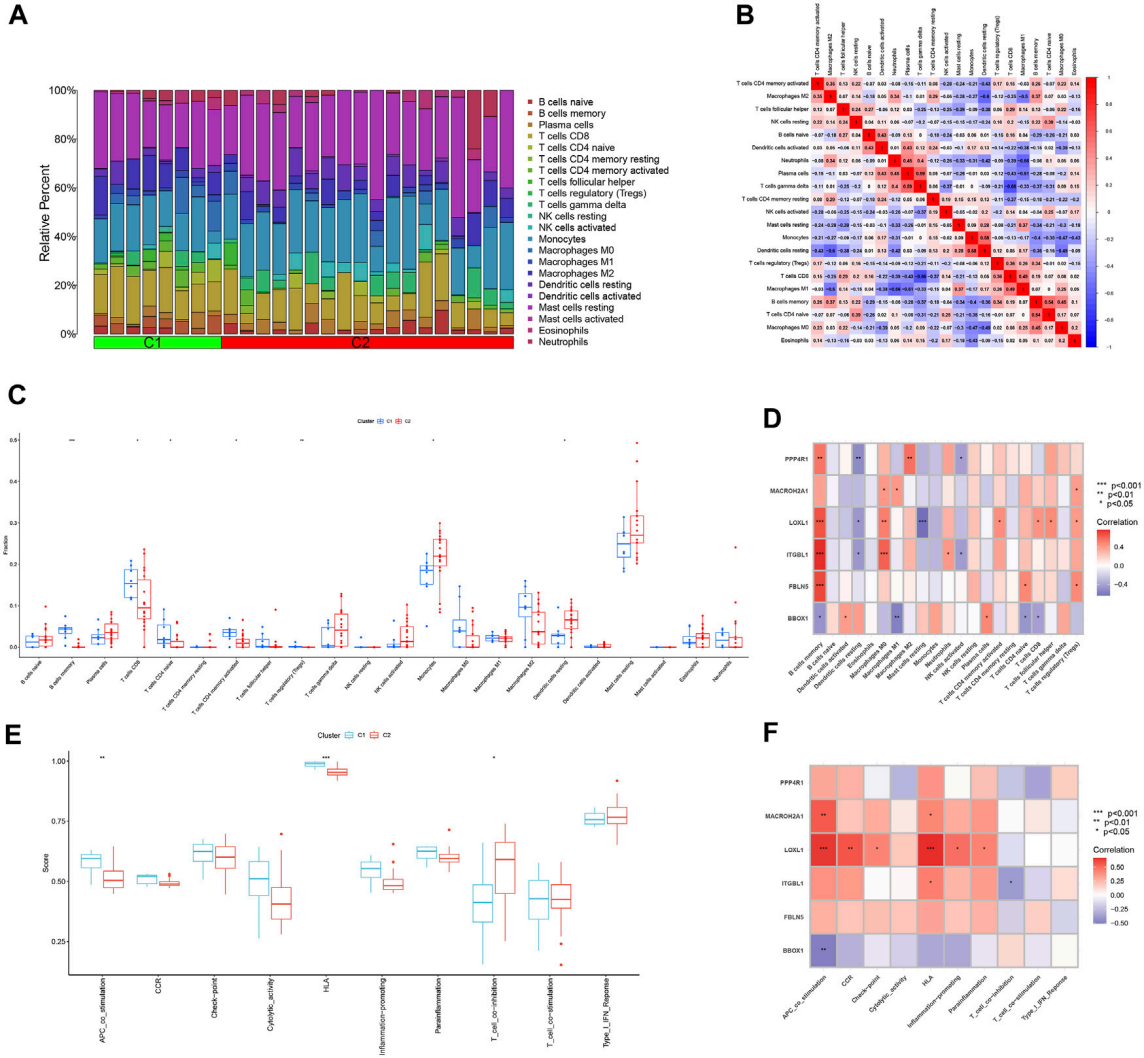
Analysis of immune infiltration between different subtypes. **(A)** Bar graph of the relative proportions of 22 immune cells between the two subtypes C1 and C2. **(B)** Heat map of the correlation between 22 immune cells. **(C)** Box line plot of expression differences of immune cells between two subtypes of C1 and C2. **(D)** Heat map of correlation between feature genes and 22 immune cells. **(E)** Box line plot of expression differences between C1 and C2 subtypes for 10 immune functions. **(F)** Heat map of the correlation between the feature genes and 10 immune functions. *p < 0.05, **p < 0.01, ***p < 0.001.

The ratio of 22 immune cells between the two subtypes C1 and C2 was evaluated using the CIBERSORT algorithm and the results are presented as bar graphs (Figure 10A). Correlation analysis between immune cells showed a positive correlation between T cells gamma delta and Plasma cells (*r* = 0.59) and a negative correlation between T cells gamma delta and T cells CD8 (*r* = −0.66) (Figure 10B). The difference in the amount of immune cell infiltration between the C1 and C2 subtypes, after removing results that were not statistically significant, showed that in the C1 subtype B cells memory, T cells CD8, T cells CD4 naïve, T cells CD4 memory activated and T cells regulatory (Tregs) expression was higher than that of the C2 subtype. In contrast, the expression of Monocytes and Dendritic cells resting was higher in C2 subtype than in C1 subtype (Figure 10C). The correlation between the feature genes and 22 immune cells showed that FBLN5, ITGBL1, LOXL1, MACROH2A1 and PPP4R1 were positively correlated with all five immune cell correlations that were highly expressed in the C1 subtype and negatively correlated with all immune cells that were highly expressed in the C2 subtype. In contrast, BBOX1 was positively correlated with both immune cell correlates highly expressed in the C2 subtype and negatively correlated with the vast majority of immune cells highly expressed in the C1 subtype (Figure 10D). Differences in 10 immune functions between C1 and C2 subtypes, after removing results that were not statistically significant, showed that APC_co_stimulation and HLA expression were higher in C1 subtype than in C2 subtype. T_cell_co-inhibition expression was higher in C2 subtype than in C1 subtype (Figure 10E). The correlation between feature genes and immune function showed that FBLN5, ITGBL1, LOXL1, MACROH2A1 and PPP4R1 were positively correlated with both immune function correlations of high expression in C1 subtype and negatively correlated with immune function of high expression in C2 subtype. In contrast, BBOX1 was positively correlated with the immune function correlation of the highly expressed C2 subtype and negatively correlated with the two immune functions of the highly expressed C1 subtype (Figure 10F).

## 4 Discussion

Hepatitis B virus infection is a major challenge to global public health. According to the World Health Organization, approximately 2 billion people worldwide are infected with hepatitis B virus, of which 257 million have CHB (Ho et al., 2020). A large number of people with CHB will progress to HBV-LC, and more than 700,000 people die each year due to HBV-LC (Kobashi et al., 2011). And the risk of progression to HCC is much greater in HBV-LC patients than in non-cirrhotic CHB patients (Do et al., 2014; Zhai et al., 2023). Therefore, the search for effective diagnostic biomarkers of HBV-LC is of great clinical value to improve its prognosis and to explore the underlying mechanisms of its formation. Cirrhosis during the transformation of CHB to HBV-LC is the result of cytopathic adaptation, where liver parenchymal cells and non-parenchymal cells undergo functional changes to adapt to the disrupted liver microenvironment (Bi and Ge, 2014; Dash et al., 2020; Meng et al., 2022). Therefore, this study attempts to screen diagnostic biomarkers of HBV-LC by high-throughput sequencing technology with bioinformatics approach and to investigate the changes that occur during the progression of CHB to HBV-LC. Hepatitis B virus infection will cause various immune responses to occur, and immune infiltration plays an important role in the progression of liver fibrosis (Zaki et al., 2022). In order to better investigate HBV-LC clinically, this study also attempted to subtype HBV-LC using the screened feature genes and to analyze the immune cell infiltration and immune function of different subtypes of HBV-LC to investigate the severity of the disease in patients with different subtypes.

In our study, 36 DEGs were identified by differential analysis from the gene expression matrix file of CHB and HBV-LC, of which 30 were upregulated and 6 were downregulated in expression. The WGCNA method was used to screen for highly correlated co-expressed genes with HBV-LC, of which 30 genes with the same DEGs were used as candidates. For further screening of diagnostic biomarkers, three machine learning algorithms, SVM-RFE, RF and LASSO logistic regression, were used. Six genes, BBOX1, LOXL1, MACROH2A1, ITGBL1, FBLN5 and PPP4R1, were finally identified as potential diagnostic biomarkers for HBV-LC. We next performed ANN model construction and validation using these 6 genes. the ROC results showed that these 6 feature genes have good accuracy and generalizability for the diagnosis of HBV-LC.

Differential analysis was conducted to discern gene expression variations between CHB and HBV-LC conditions. The outcomes unveiled a distinct pattern wherein BBOX1 exhibited an upregulation in CHB but a downregulation in HBV-LC expression. Conversely, the remaining five genes exhibited an upregulation in HBV-LC expression while being downregulated in CHB expression. Notably, BBOX1 has been identified in vital organs such as the liver, kidney, and brain. Functionally, it is associated with L-carnitine synthesis, encoding Gamma-butyrobetaine hydroxylase—a pivotal enzyme governing carnitine synthesis. This enzyme is notably vital due to its role in L-carnitine production, a pivotal molecule in the realm of fatty acid metabolism (Rigault et al., 2006). The findings suggest a potential reduction in hepatic fatty acid metabolism during the onset of HBV-LC. Moreover, studies have demonstrated that a decrease in the expression of BBOX1 antisense RNA 1 (BBOX1-AS1) corresponds to diminished cellular viability and proliferation (Zhao et al., 2022), and low BBOX1 expression serves as a prognostic biomarker for clear cell renal cell carcinoma (RCC) (Kim et al., 2023). Regrettably, the existing literature does not encompass any articles pertaining to the association between BBOX1 and cirrhosis. In contrast, LOXL1 is classified within the LOX gene family, encoding Lysyl oxidase-like 1. Portal fibroblasts are responsible for the synthesis of LOX and LOXL1, which subsequently undergo cross-linking with elastin. Notably, their enzymatic activity undergoes augmentation during the transition of fibroblasts into myofibroblasts (Li et al., 2007; Perepelyuk et al., 2013). Therefore, upregulation of LOXL1 gene expression will promote the development of liver fibrosis and even cirrhosis. And it has been shown that LOX family has been identified as a therapeutic target for liver fibrosis (Chen et al., 2020). MACROH2A1 is a gene encoding an isoform of histone H2A protein. Both isoforms of MACROH2A1 (MACROH2A1.2 and MACROH2A1.2) have been shown to increase with age in both rodent and human livers and are strong immunohistochemical markers of human cirrhosis and HCC (Macias et al., 2021). ITGBL1 is a gene encoding a beta-integrin-related protein, and ITGBL1 protein is an extracellular matrix protein (Sun et al., 2016). ITGB1 has been shown to be a key regulator of fibrosis in patients with hepatitis B virus-associated liver fibrosis (HBV-LF) by analyzing the genetic profile of these patients (Wang et al., 2017). FBLN5 encodes Fibulin-5, which belongs to the Fibulin family of secreted extracellular matrix proteins, and is a target of TGF-β in fibroblasts and endothelial cells (Lee and Schiemann, 2011). Therefore, upregulation of FBLN5 expression can also promote HBV-LC formation. PPP4R1 encodes serine/threonine protein phosphatase 4 regulatory subunit 1. No explicit investigation has established a direct link between PPP4R1 and the onset of cirrhosis. Nevertheless, in a particular study, heightened expression of CCDC6 exhibited a robust correlation with unfavorable prognoses in hepatobiliary cancer cases. Furthermore, a significant association was observed between the CCDC6 gene, its corresponding protein, and PPP4R1 (Wu et al., 2022). The incidence of HCC was significantly higher in HBV-LC patients than in CHB non-cirrhotic patients (Do et al., 2014; Zhang et al., 2023). Therefore, there should also be a relationship between the upregulation of PPP4R1 expression and the formation of cirrhosis.

In addition, we performed single-gene GSEA analysis of the feature genes. The results showed that BBOX1 was highly expressed in bile secretion function, and the downregulation of BBOX1 expression in the presence of HBV-LC reduced bile secretion from hepatocytes, which led to a weakened digestion and absorption of fat by the organism. This view is consistent with the aforementioned decrease in fatty acid metabolic function due to BBOX1 downregulation. Both FBLN5 and PPP4R1 were significantly enriched in the Phosphatidylinositol signaling system, indicating that these two genes could be involved in the conversion of CHB to HBV-LC by regulating the signaling process of phosphatidylinositol. While three genes, ITGBL1, LOXL1 and MACROH2A1, were highly expressed in the Proteasome pathway, PPP4R1 was highly expressed in the p53 signaling pathway. Both pathways regulate cell cycle progression, thus suggesting that these four genes may be involved in the conversion of CHB to HBV-LC by regulating cell cycle progression (Do et al., 2014; Hernández Borrero and El-Deiry, 2021). Moreover, ITGBL1, LOXL1 and PPP4R1 are all significantly enriched in the Ribosome pathway, so these three genes may also influence the formation of cirrhosis by regulating ribosome function.

22 immune cell infiltration analyses were performed. The results showed that the expression of B cells memory, T cells CD8, T cells CD4 naive, T cells CD4 memory activated and T cells regulatory (Tregs) was higher in C1 subtype than in C2 subtype. The expression of Monocytes and Dendritic cells resting was higher in the C2 subtype than in the C1 subtype. The correlation between the feature genes and 22 immune cells showed that ITGBL1, LOXL1 and FBLN5 were positively correlated with five immune cells of C1 subtype with high infiltration, and negatively correlated with two immune cells of C2 subtype with high infiltration. In contrast, BBOX1 was positively correlated with both immune cell correlations of C2 subtype hyperinfiltrative and negatively correlated with all five immune cells of C1 subtype hyperinfiltrative. The degree of damage in hepatitis B virus-infected patients correlated with the degree of immunity of the patients. Hepatitis B virus is a non-cellular lesion on infected hepatocytes, and the resulting liver damage is caused by an auto-specific immune response. T cells not only clear hepatitis B virus, but also cause damage to damaged hepatocytes (Guidotti and Chisari, 2006). Therefore, the higher the T-cell activity, the more severe the liver damage will be, and the more severe the cirrhosis will be in order to repair the liver damage. The results of immune cell infiltration show that the C1 subtype has high expression of multiple T cells, while the C2 subtype has low expression of dendritic cells as antigen-presenting cells (APC). From the results of immune cell infiltration analysis, the extent of disease in patients with C1 subtype HBV-LC will probably be heavier than that of C2 subtype.

10 immune function analyses were performed. The results showed that APC_co_stimulation and HLA expression were higher in the C1 subtype than in the C2 subtype after removal of statistically insignificant results. T_cell_co-inhibition expression was higher in the C2 subtype than in the C1 subtype. The correlation between feature genes and immune functions showed that ITGBL1, LOXL1 and FBLN5 were positively correlated with both immune functions highly expressed in the C1 subtype and negatively correlated with immune functions highly expressed in the C2 subtype. In contrast, BBOX1 was positively correlated with the immune function correlation of high expression in the C2 subtype and negatively correlated with both immune functions of high expression in the C1 subtype. Human leukocyte antigen (HLA) is the gene encoding the MHC protein, and T cell activation and differentiation requires binding to MHC II molecules and costimulatory molecules on antigen-presenting cells (APCs) (Bertram et al., 2004; Mott et al., 2014; Zhao et al., 2022; Gong et al., 2022; Xiong et al., 2023). The C1 subtype is highly expressed in APC_co_stimulation and HLA functions, while the C2 subtype is highly expressed in T_cell_co-inhibition functions. Therefore, HBV-LC patients with C1 subtype will have significantly higher T-cell activity than those with C2 subtype. Therefore, the degree of disease may be heavier in the C1 subtype than in the C1 subtype by immune function analysis. Combined with the analysis of immune cell infiltration and immune function between different subtypes of HBV-LC, both concluded that patients with subtype C1 may be sicker than those with subtype C2.

Although our study elucidates the possible mechanisms of CHB conversion to HBV-LC and provides clinically meaningful options for the early diagnosis and treatment of HBV-LC patients, our study still has some degree of limitations. First, this study was a retrospective study and therefore needs to be validated by future prospective studies. Second, the sample size of this study was limited and was obtained from the GEO database. Third, although our study identified HBV-LC associated genes and elucidated some of the mechanisms, further experiments are needed to elucidate the more in-depth mechanisms of these signature genes in the disease.

## 5 Conclusion

By using bioinformatics analysis, this study identified six feature genes, BBOX1, LOXL1, MACROH2A1, ITGBL1, FBLN5, and PPP4R1, involved in the conversion of CHB to HBV-LC, providing important information to elucidate the potential molecular mechanism of CHB progression to HBV-LC. This study also screened four genes ITGBL1, LOXL1, FBLN5, and BBOX1 associated with HBV-LC subtype delineation by analysis of feature genes. and explored the possible disease severity between patients with different subtypes at the immune level. Our findings will probably contribute to the design of better diagnostic and therapeutic approaches for HBV-LC based on molecular mechanisms.

## Data Availability

The original contributions presented in the study are included in the article/Supplementary material, further inquiries can be directed to the corresponding authors.
